# An investigation of the possibility of chemosensitization by clinically achievable concentrations of misonidazole.

**DOI:** 10.1038/bjc.1983.26

**Published:** 1983-02

**Authors:** P. R. Twentyman, P. Workman

## Abstract

Experiments have been carried out both in vitro and in vivo to examine the possibility of chemosensitization by misonidazole (MISO) at concentrations which are achievable in the clinic. Using multicellular tumour spheroids in vitro we found that a 16 h pre-incubation with 100 micrograms ml-1 MISO under hypoxic conditions led to a considerable enhancement of sensitivity to melphalan (MEL) but not to CCNU. Pre-incubation for 16 h under hypoxia alone also produced a degree of sensitization to MEL, but there was no effect of oxic pre-incubation with MISO. In vivo experiments using the KHT or RIF-1 tumours in C3H mice were designed so that repeated administration of MISO maintained blood concentrations of around 100 micrograms ml-1 for either 7 h or 16 h. For the 7 h regime, cytotoxic drugs were administered at the 4 h point. In most experiments the tumour response to MEL, cyclosphosphamide (CTX), chlorambucil or CCNU was no greater in mice receiving multiple MISO than in mice receiving multiple injections of a balanced salt solution. In the occasional experiment where there was an apparent increase in response, the effect was only small (dose modifying factor less than 1.5). For the 16 h regime the effect was studied of administering CTX (100 mg kg-1) at various times during the regime. There was a clear trend towards increased CTX response in mice receiving multiple MISO compared with controls. There was, however, no clear tendency for the effect to increase with length of MISO pre-exposure.


*Br. J. Cancer* (1983) **47**, 187–194

### An investigation of the possibility of chemosensitization by clinically achievable concentrations of misonidazole

P.R. Twentyman & P. Workman

*MRC Clinical Oncology and Radiotherapeutics Unit, MRC Centre, Hills Road, Cambridge, CB2 2QH.*

**Summary** Experiments have been carried out both *in vitro* and *in vivo* to examine the possibility of chemosensitization by misonidazole (MISO) at concentrations which are achievable in the clinic. Using multicellular tumour spheroids *in vitro* we found that a 16 h pre-incubation with $100\,\mu g\,ml^{-1}$ MISO under hypoxic conditions led to a considerable enhancement of sensitivity to melphalan (MEL) but not to CCNU. Pre-incubation for 16 h under hypoxia alone also produced a degree of sensitization to MEL, but there was no effect of oxic pre-incubation with MISO. *In vivo* experiments using the KHT or RIF-1 tumours in C3H mice were designed so that repeated administration of MISO maintained blood concentrations of around $100\,\mu g\,ml^{-1}$ for either 7 h or 16 h. For the 7 h regime, cytotoxic drugs were administered at the 4 h point. In most experiments the tumour response to MEL, cyclosphosphamide (CTX), chlorambucil or CCNU was no greater in mice receiving multiple MISO than in mice receiving multiple injections of a balanced salt solution. In the occasional experiment where there was an apparent increase in response, the effect was only small (dose modifying factor $<1.5$). For the 16 h regime the effect was studied of administering CTX ($100\,mg\,kg^{-1}$) at various times during the regime. There was a clear trend towards increased CTX response in mice receiving multiple MISO compared with controls. There was, however, no clear tendency for the effect to increase with length of MISO pre-exposure.

The electron-affinic agent, misonidazole (MISO), has been developed extensively as a radiation sensitizer of hypoxic cells (Adams, 1977). More recently, two separate lines of investigation have suggested that this agent may be of clinical value in producing selective sensitization of tumour cells to some cytotoxic drugs. It was shown by Stratford *et al.* (1980) that pre-incubation of Chinese hamster ovary (CHO) cells with MISO under hypoxic (but not oxic) conditions led to an enhanced sensitivity to nitrogen mustard, melphalan and cis-platinum, and this effect has been confirmed by Twentyman (1982) using growth delay in EMT6 tumour spheroids as the response endpoint. At the same time, a number of *in vivo* studies (Rose *et al.*, 1980; Clement *et al.*, 1980; Tannock, 1980; Law *et al.*, 1981; Twentyman, 1981; Martin *et al.*, 1981; Siemann, 1981) have found that administration of MISO to mice at or around the time of cytotoxic drug administration can increase the anti-tumour effect of the cytotoxic drug.

In some of these studies a therapeutic gain is claimed in that the enhancement of the cytotoxic drug effect against the tumour is greater than that seen using a variety of normal tissue response endpoints.

These experimental investigations, both *in vitro* and *in vivo*, have, however, been carried out using concentrations or doses of MISO which are much higher than those normally attainable in the clinic. The *in vitro* studies of Stratford *et al.* (1980) and Twentyman (1982) both used a MISO concentration of 5 mM ($1000\,\mu g\,ml^{-1}$) and a pre-incubation time of 2–5 h. The various *in vivo* experiments in the mouse have used MISO doses in the range $1.5$–$5\,mM\,kg^{-1}$ ($0.3$–$1\,g\,kg^{-1}$). These would be expected to produce peak plasma concentrations of 1.5–5 mM ($300$–$1000\,\mu g\,ml^{-1}$). In contrast the largest single dose of MISO which is usually given in clinical practice ($3\,g\,m^{-2}$) produces peak plasma concentrations of only 0.5–0.75 mM ($100$–$150\,\mu g\,ml^{-1}$) (for review see Workman, 1980). The plasma half-life of MISO in humans, however, is 10–20 times longer than in the mouse (Workman, 1980) and therefore the relative contributions to chemosensitization of peak plasma level and of exposure time is clearly a matter of importance.

In this paper we describe experiments designed to examine, both *in vitro* and *in vivo* the possibility of chemosensitization by clinically achievable concentrations of MISO. A study with similar objectives, recently reported by Brown & Hirst (1982) has produced encouraging *in vivo* data.

### Materials and methods

### *Multicellular tumour spheroids*

The growth conditions and experimental procedures for *in vitro* experiments with spheroids of the EMT6/Ca/VJAC mouse tumour line were as

Received 12 July 1982, accepted 4 October 1982.

0007-0920/83/020187-08 $02.00

© The Macmillan Press Ltd., 1983

previously described (Twentyman, 1982). Briefly, spheroids were grown in agar-coated flasks to a diameter of 250 $\mu$ and then transferred to 100 ml-spinner culture flasks for pre-incubation. In these experiments, pre-incubation at 37°C was either in the presence or absence of MISO (100 $\mu g\,ml^{-1}$) for 16 h under either oxic or hypoxic conditions. Hypoxia was achieved by passing nitrogen with 5% $CO_2$ (<10 pt $10^{-6}$ $O_2$; British Oxygen Co.) into the spinner vessels at a rate of 750–1000 ml $min^{-1}$. After pre-incubation and rinsing, spheroids were exposed to graded concentrations of either melphalan (MEL) or CCNU for 1 h in glass tubes with intermittent agitation. After this time, spheroids were again rinsed and transferred to individual wells on 96-well plastic multidishes. Successive measurements of spheroid diameter were made and growth curves constructed as previously described (Twentyman, 1982).

*Mice and tumours*

The mice used in these studies were inbred C3H/He supplied by OLAC. Females were used in most experiments, but males were used occasionally. Mice entered experiments at age 12–16 weeks and weighed 20–28 g.

Tumours used were the KHT and RIF-1 sarcomas, both of which originated in C3H/Km mice at Stanford University, California, and which have been previously described (Kallman *et al.*, 1967; Twentyman *et al.*, 1980). The methods used for tumour cell inoculation into the gastrocnemius muscle of the hind limb and subsequent measurement of tumour growth, including conversion of leg measurement to tumour weight, have also been described (Twentyman *et al.*, 1979). The endpoint of growth delay was calculated from the time taken for individual tumours to reach 4 × their initial treatment volume. Tumours were treated in the size range 300–600 $mm^3$. Eight to 12 mice were used in each treatment group.

*Drugs*

Cytotoxic drugs for *in vivo* use were obtained, prepared and administered as shown in Table I. For *in vitro* use, MEL was dissolved in acidified ethanol and CCNU in absolute ethanol so that a volume of 0.03–0.2 ml could then be added to 10 ml of medium to give the required final concentrations. MISO for *in vitro* use was dissolved in Hanks' balanced salt solution (HBSS) at a concentration of 2.5 mg $ml^{-1}$ and then diluted 1:25 for pre-incubation. For *in vivo* use, MISO was dissolved in HBSS so that either the loading dose or "top-up" doses could be administered in a volume of 0.01 ml $g^{-1}$. In order to achieve and maintain blood MISO concentrations at or around 100 $\mu g\,ml^{-1}$, a loading dose of 0.24 mM $kg^{-1}$ was given initially followed by subsequent doses of 0.15 mM $kg^{-1}$ at 30 min intervals. These doses were based on data from Brown (1982) and our own pharmacokinetic data. All injections were via the i.p. route, and mice not receiving MISO were given appropriate volumes of HBSS as a control. Concentrations of MISO and its metabolite desmethylmisonidazole (DEMIS) in blood were monitored during all

**Table I** Cytotoxic drugs—Preparation and administration *in vivo*

| *Drug* | *Supplier* | *Preparation* | *Administration volume* |
|---|---|---|---|
| cyclophosphamide (CTX) | Ward Blenkinsopp | Dissolve in HBSS | 0.005 –0.02 ml $g^{-1}$ |
| melphalan (MEL) | Chester Beatty Research Institute | Dissolve in acidified ethanol. Dilute 1:10 in propylene glycol—$K_2HPO_4$ buffer, final pH 7.4 | 0.01 ml $g^{-1}$ |
| 1-(2-chloroethyl)-3-cyclohexyl-1-nitrosourea (CCNU) | U.S. National Cancer Institute | Dissolve in absolute ethanol. Dilute 1:20 in 0.5% carboxymethyl cellulose/Hanks | 0.005 –0.015 ml $g^{-1}$ |
| chlorambucil (CHL) | Chester Beatty Research Institute | (1) Dissolve in absolute ethanol. Dilute 1:10 in arachis oil B.P. or (2) As for melphalan | 0.01 ml $g^{-1}$ |

experiments using tail-vein sampling (Workman, 1979) and reversed-phase high-performance liquid chromatography analysis (HPLC) (Workman *et al.*, 1978). Studies were carried out to confirm that tail-vein blood concentrations were identical to those obtained after cardiac puncture.

Two regimes of multiple MISO administration were used. In protocol A, administration continued up to 7 h, and the cytotoxic drugs were given between the 3.5 h and 4 h injections. In protocol B (only used for CTX), multiple MISO administration was extended to 16 h, and CTX was given either immediately before the initial dose, or immediately before the 4, 8, 12 or 16 h subsequent doses. Neither of these regimes resulted in a significant fall in mouse body temperature.

### Results

### *In vitro*

The results of spheroid growth delay experiments are shown in Figures 1 and 2. In each case, any

**Figure 1** Growth delay in small (250 μ) EMT6 spheroids induced by melphalan after various conditions of pre-incubation for 16 h. ○ oxic − MISO △ oxic + MISO ● hypoxic − MISO ▲ hypoxic + MISO.

**Figure 2** Growth delay in small (250 μ) EMT6 spheroids induced by CCNU. Symbols as Figure 1.

growth delay due to pretreatment alone has been subtracted before the points were calculated. Mean values for 5 experiments under each condition were 0.0, 0.4 and 1.0 days for MISO alone, hypoxia alone and MISO plus hypoxia respectively. It may be seen that for MEL (Figure 1), pretreatment with MISO under hypoxic conditions leads to considerable sensitization. There is also some apparent effect of pretreatment under hypoxia without MISO, but to a much smaller extent. Dose modification factors may be calculated from the best lines fitted by eye to the points. For growth delays of 5 days and 8 days respectively the values are 1.27 and 1.61 for hypoxia alone and 2.76 and 2.87 for hypoxia + MISO. In contrast, the results for CCNU (Figure 2) indicate little or no effect of pretreatment under hypoxic conditions either with or without MISO.

### *In vivo*

The blood concentrations of MISO obtained in the 2 experiments using the 16 h protocol are shown in Figure 3. It may be seen that the regime was successful in maintaining steady-state blood concentrations close to the intended value of 100 μg ml$^{-1}$. Concentrations of the desmethyl

**Figure 3** Plasma levels of MISO and the metabolite DEMIS during the two 16h multiple-MISO injection experiments. Open and closed symbols are 2 separate experiments. Error bars represent ±2 s.e. of the mean for groups of 4–5 mice.

metabolite DEMIS were also constant but at the lower level of about $20\,\mu g\,ml^{-1}$. Steady state concentrations of MISO and DEMIS obtained in individual experiments using the 7h protocol are summarised in Table II.

Results obtained for the response to cytotoxic drugs injected 3.5–4h into the 7h protocol are shown in Tables III–IV. For CTX (Table III) the results are largely negative in that the growth delay for a given CTX dose is not significantly different in mice receiving MISO from that in mice receiving HBSS. In one experiment (i.e. Expt. B, $100\,mg\,kg^{-1}$) the difference is significant but the additional effect in the MISO treated group is less than that produced by increasing the CTX dose from 100 to $150\,mg\,kg^{-1}$ in control mice. For MEL (Table IV), the results are negative in 2/3 experiments. In experiment B, however, enhancement of MEL by a factor approaching 1.5× was seen (i.e. MEL

**Table II** Steady-state blood concentrations of MISO and its metabolite DEMIS in experiments using the 7h protocol

| | *Steady-state blood concentration* ($\mu g\,ml^{-1}$) | |
|---|---|---|
| *Experiment* | *MISO* | *DEMIS* |
| A | 83.8 (3.4) | 19.3 (2.1) |
| B | 93.4 (12.5) | 21.2 (2.1) |
| C | 95.4 (13.0) | 29.2 (1.3) |
| D | 91.8 (5.3) | 18.9 (1.7) |
| F | 108.5 (9.9) | 15.8 (3.9) |
| G | 113.3 (22.8) | 24.1 (3.2) |
| H | 113.0 (9.8) | 31.1 (3.4) |

Blood concentrations were normally measured at hourly intervals from 1–7h, with 4–5 mice per group. The results presented are the overall means of the individual group mean values from 2–7h ($n=6$, except for Experiment G where $n=4$) with 2 s.e. in parentheses.

**Table III** Growth delay in RIF-1 tumours treated with CTX

| | | *Time to* 4× *treatment volume* (*days*) | |
|---|---|---|---|
| *Experiment* | *CTX dose* ($mg\,kg^{-1}$) | *Multi HBSS pretreatment* | *Multi MISO pretreatment* |
| A | 0 | 3.7 (2.7– 5.1) | 3.7 (2.8– 5.0) |
| | 100 | 11.9 (10.8–13.1) | 13.2 (12.2–14.3) |
| B | 0 | 7.2 (6.1– 8.5) | 7.0 (6.2– 8.0) |
| | 100 | 16.1 (15.1–17.1) | 20.8 (19.1–22.6) |
| | 150 | 23.6 (20.6–27.0) | — |
| C* | 0 | 8.7 (7.7– 9.8) | 8.0 (7.3– 8.8) |
| | 50 | 12.1 (10.8–13.6) | 14.6 (13.7–15.5) |
| | 100 | 26.7 (22.0–32.2) | 27.9 (22.4–34.7) |
| | 150 | 36.4 (32.7–40.7) | 35.1 (30.3–40.7) |
| D | 0 | 4.8 (4.2– 5.6) | 5.1 (4.3– 6.0) |
| | 100 | 15.2 (14.6–15.8) | 16.4 (15.1–17.8) |
| | 150 | 21.4 (19.1–24.1) | — |

Values given are geometric means for groups of 8–12 mice. Figures in parentheses are 2 s.e. limits.

*The control tumour growth rates and CTX growth delays are atypically long in this experiment. In nearly all experiments with the RIF-1 tumour, the time to 4× treatment volume for control tumours lies in the region 4–7 days.

**Table IV** Growth delay in RIF-1 tumours treated with melphalan

| Experiment | *MEL dose* (*mg kg*$^{-1}$) | *Time to* 4 × *treatment volume* (*days*) *Multi HBSS pretreatment* | *Multi MISO pretreatment* |
|---|---|---|---|
| A | 0 | 3.7 (2.7– 5.1) | 3.7 (2.8– 5.0) |
| | 10 | 9.1 (7.8–10.6) | 10.1 (9.0–11.2) |
| B | 0 | 7.2 (6.1– 8.5) | 7.0 (6.2– 8.0) |
| | 8 | 11.0 (9.8–12.5) | 14.2 (13.0–15.5) |
| | 12 | 15.6 (14.5–16.9) | |
| D | 0 | 4.8 (4.2– 5.6) | 5.1 (4.3– 6.0) |
| | 8 | 8.4 (7.1– 9.8) | 8.8 (7.8–10.0) |
| | 12 | 11.7 (9.8–14.1) | |

Vales given are geometric means for groups of 8–12 mice.
Figures in parentheses are 2 s.e. limits.

**Table V** Growth delay in RIF-1 tumours treated with chlorambucil

| Experiment | *CHL dose* (*mg kg*$^{-1}$) | *Time to* 4 × *treatment volume* (*days*) *Multi HBSS pretreatment* | *Multi MISO pretreatment* |
|---|---|---|---|
| A | 0 | 3.7 (2.7– 5.1) | 3.7 (2.8– 5.0) |
| | 7.5 | 6.6 (5.7– 7.3) | 7.3 (6.3– 8.6) |
| | 7.5 | — | 10.4 (9.6–11.3)* |
| D | 0 | 4.8 (4.2– 5.6) | 5.1 (4.3– 6.0) |
| | 10 | 9.0 (7.9–10.2) | 12.7 (11.9–13.5) |
| | 15 | 11.9 (11.4–12.5) | — |
| F | 0 | 6.2 (5.4– 7.2) | 6.5 (5.5– 7.8) |
| | 8 | 8.5 (7.4– 9.6) | 9.7 (8.8–10.6) |
| | 12 | 10.2 (8.8–11.8) | 11.8 (10.7–13.1) |
| | 16 | 12.8 (11.2–14.6) | 14.2 (13.1–15.3) |

*Single dose MISO (2.5 mM kg$^{-1}$) 30 min before CHL.
Values given are geometric means for groups of 8–12 mice.
Figures in parentheses are 2 s.e. limits.

8 mg kg$^{-1}$ + MISO produces a growth delay nearly equal to that given by MEL 12 mg kg$^{-1}$ alone).

The data for CHL (Table V) are again similar with little or no enhancement being shown in 2/3 experiments. In experiment D, however, an enhancement of slightly greater than 1.5 was produced. It may be seen that in experiment A a direct comparison was made of multi low-dose MISO with the effect of a large single dose of MISO given 30 min before the CHL. The negative result for the low dose protocol contrasts with the clear enhancement produced by the single dose.

For CCNU (Table VI) neither experiment showed a significant enhancement by multiple MISO.

In the light of these mainly negative results, 2 experiments were carried out with CTX in combination with a 16 h regime of MISO administration. The results of the first experiment are shown in Figure 4. It may be seen that at 2 times (i.e. 4 and 16 h) the values for CTX with MISO are significantly greater than those for CTX with HBSS. At the other times, the values are also greater but not significantly so. If all the groups are combined (i.e. irrespective of relative time of administration) the growth delay in mice receiving HBSS + CTX was 11.0 ± 2.2 days and that in mice receiving MISO + CTX was 16.6 ± 2.3 days. In the same experiment, we also looked at the effect of large single doses of MISO, given 30 min before CTX (100 mg kg$^{-1}$) and obtained growth delays of 16.6 ± 2.5 days for CTX + 5 mM kg$^{-1}$ of MISO and 15.9 ± 4.9 days for CTX + 2.5 mM kg$^{-1}$ MISO

**Table VI** Growth delay in KHT tumours treated with CCNU

| Experiment | *CCNU dose* ($mg\ kg^{-1}$) | *Time to 4 × treatment volume (days)* Multi HBSS pretreatment | Multi MISO pretreatment |
|---|---|---|---|
| G | 0 | 2.5 (2.3– 2.8) | 2.8 (2.5– 3.3) |
| | 10 | 5.8 (4.1– 8.4) | 7.4 (6.2– 8.8) |
| | 20 | 15.7 (11.0–22.0) | 17.1 (13.8–21.0) |
| | 30 | 24.0 (21.0–28.0) | — |
| H | 0 | 2.4 (2.2– 2.7) | 2.5 (2.2– 2.8) |
| | 10 | 5.1 (3.5– 7.4) | 7.8 (5.1–11.7) |
| | 20 | 16.9 (15.4–18.5) | 17.9 (17.3–18.6) |

Values given are geometric means for groups of 8–12 mice. Figures in parentheses are 2 s.e. limits.

**Figure 4** Growth delay in RIF-1 tumours induced by CTX ($100\ mg\ kg^{-1}$) injected at various times during the 16 h regime of multiple MISO or HBSS administration. Error bars represent ±2 s.e. of the geometric mean for groups of 8–10 mice.

compared with 10.8±2.0 days for CTX alone. It would therefore appear that, in this experiment, the multiple MISO regime produced as much enhancement of CTX as a large single dose of $5\ mM\ kg^{-1}$. An exact repeat of this experiment produced the results shown in Table VII. It may be seen that although the growth delays were shorter in this experiment, the multiple MISO regime is again about as effective as a large single dose MISO ($5\ mM\ kg^{-1}$) in enhancing the effect of CTX. It does not appear, however, that either MISO regime is capable of modifying the effect of CTX ($100\ mg\ kg^{-1}$) to that produced by $150\ mg\ kg^{-1}$ of CTX alone.

**Table VII** Effect of CTX ($100\ mg\ kg^{-1}$) on the RIF-1 tumour at various times during a 16 h protocol of MISO or HBSS administration

| *Time (h)* | *Time to 4 × treatment volume (days)* Multiple HBSS | Multiple MISO |
|---|---|---|
| 0 | 15.5 (13.8–17.4) | 18.8 (16.4–21.5) |
| 4 | 18.0 (15.2–21.3) | 17.1 (15.5–18.9) |
| 8 | 12.6 (10.9–14.8) | 16.4 (13.8–19.5) |
| 12 | 13.5 (11.9–15.4) | 17.4 (15.9–19.1) |
| 16 | 15.8 (14.5–17.2) | 17.3 (15.3–19.7) |
| All times combined | 14.9 (13.9–16.0) | 17.4 (16.4–18.3) |
| Control i.e. 0 CTX | 8.9 (8.1–9.6) | 9.2 (8.1–10.6) |

The growth delay is therefore 8.2 days for MISO pretreatment compared with 6.0 days for HBSS pretreatment. In the same experiment, single dose MISO ($5\ mM\ kg^{-1}$) increased the growth delay due to CTX $100\ mg\ kg^{-1}$) from 6.0 days to 9.1 days, compared with 11.7 days for CTX ($150\ mg\ kg^{-1}$) alone.

### Discussion

The data from our *in vitro* experiments indicate that a 16-h exposure to $100\ \mu g\ ml^{-1}$ of MISO under hypoxic conditions makes spheroids much more sensitive to growth delay induced by MEL. This is not true, however, for CCNU. These results are in agreement with our earlier study (Twentyman, 1982) where there were also differences in the ability of short hypoxic pre-exposure to MISO to sensitize spheroids to these two agents. We found that dose modifying factors were higher for MEL than for CCNU, and that there was a greater tendency with MEL than with CCNU for modification to depend upon the length of the pre-exposure period.

The results for our *in vivo* experiments using a 7-h MISO protocol are disappointing. In the majority of experiments no significant increase in drug response was caused by this MISO regime. Only in a single determination for MEL and a single determination for CHL are the data compatible with a dose modification of around 1.5. The bulk of the data suggest that dose modification by a factor >1.2 is unlikely. This is clearly less than the dose-modifying factors which have been seen following large single dose MISO in the RIF-1 tumour with CTX (Twentyman, 1981; Law *et al.*, 1981) or in the KHT tumour with CCNU (Siemann, 1981; Workman and Twentyman, 1982). The results are contrary to those recently reported by Brown & Hirst (1982) who used a similar 7-h MISO protocol in the RIF-1 tumour and demonstrated clear sensitization to MEL and to CTX. Also, using different mouse tumour systems and 8 h regimes of MISO exposure, positive chemosensitization to MEL and CTX has been found by Dr. N.J. McNally (personal communication) but an absence of effect is seen by Randhawa and Denekamp (personal communication). This disparity of results may indicate that the 7–8 h exposures are close to some critical level necessary for chemosensitization.

The 7-h regime with cytotoxic drug administration at 3.5–4 h was originally chosen by Brown & Hirst (1982) as it closely resembles the clinical situation as used in radiotherapy. The peak plasma MISO concentration in man occurs at around 4 h after a single dose of $3\,g\,m^{-2}$ and does not fall much over the subsequent few hours. By giving the cytotoxic drug at this time we therefore had the advantage of a period of pre-exposure to MISO before cytotoxic drug administration, high levels at the time of drug administration, and continuing high levels during the active life of the drug. On the other hand, because of the relatively rapid fall in MISO levels in the mouse following the final MISO injection ($t\frac{1}{2} \sim 1$ h compared with $\sim 10$ h in man), the total area under the blood MISO *versus* time curve (AUC) is still considerably less for the 7 h protocol in the mouse (3 mM.h) compared with that following $3\,g\,m^{-2}$ in man (8 mM.h) (see Workman, 1980). We therefore decided to extend the multiple injection regime in the mouse to 16 h (which does give an AUC of $\sim$8 mM.h) in order to closely equate the total MISO exposures.

Our data for CTX indicate that this 16 h protocol produces more enhancement than the 7 h protocol. However, there is a complete absence of time-dependency in the effect, i.e. the time of CTX administration with respect to the 16 h MISO regime does not appear to be critical. This is not the result which would have been expected if the *in vivo* effect were due solely to a progressive depletion of thiols with time and may indicate that a number of contributing mechanisms are involved as found *in vitro* (Taylor *et al.*,1982).

In conclusion, therefore, our *in vitro* studies indicate that clinically achievable MISO concentrations cause considerable sensitization of spheroids to MEL but not to CCNU. Our *in vivo* results are mostly negative for a 7 h MISO protocol, but positive with CTX for a 16 h regime.

We thank Jane Donaldson, Jill Shaw, Kate Smith, Nancy Smith, Michael Walton and Karen Wright for their excellent technical assistance.

### References

ADAMS, G.E. (1977). Hypoxic cell radiosensitizers for radiotherapy. In *Cancer: A Comprehensive Treatise, Vol. 6,* (Ed. Becker) New York: Plenum Press, p. 181.

BROWN, J.M. (1982). Mechanisms of cytotoxicity and chemosensitization by nitroimidazoles. *Int. J. Radiat. Oncol. Biol. Phys.*, **8,** 675.

BROWN, J.M. & HIRST, D.G. (1982). Effect of clinical levels of misonidazole on the response of tumour and normal tissues in the mouse to alkylating agents. *Br. J. Cancer* **45,** 700.

CLEMENT, J.J., GORMAN, M.S., WODINSKY, I., CATANE, R. & JOHNSON, R.K. (1980). Enhancement of antitumour activity of alkylating agents by the radiation sensitizer misonidazole. *Cancer Res.*, **40,** 4165.

KALLMAN, R.F., SILINI, G. & VAN PUTTEN, L.M. (1967). Factors influencing the quantitative estimation of the *in vivo* survival of cells from solid tumours. *J. Natl. Cancer Inst.*, **39,** 539.

LAW, M.P., HIRST, D.B. & BROWN, J.M. (1981). The enhancing effect of misonidazole on the response of the RIF-1 tumour to cyclophosphamide. *Br. J. Cancer,* **44,** 208.

MARTIN, W.M.C., MCNALLY, N.J. & DERONDE, J. (1981). The potentiation of cyclophosphamide cytotoxicity by misonidazole. *Br. J. Cancer,* **43,** 756.

ROSE, C.M., MILLAR, J.L., PEACOCK, J.H. & STEPHENS, T.C. (1980). The effect of misonidazole on *in vivo* tumour cell kill in Lewis lung carcinoma treated with melphalan or cyclophosphamide. In *Radiation Sensitizers—Their Use in the Clinical Management of Cancer.* (Ed. Brady), New York: Masson, p. 250.

SIEMANN, D.W. (1981). The *in vivo* combination of the nitroimidazole misonidazole and the chemotherapeutic agent CCNU. *Br. J. Cancer,* **43,** 367.

STRATFORD, I.J., ADAMS, G.E., HORSMAN, M.R. & 4 others. (1980). The interaction of misonidazole with radiation, chemotherapeutic agents or heat. A preliminary report. *Cancer Clin. Trials,* **3,** 231.

TAYLOR, Y.C., BUMP, E.A. & BROWN, J.M. (1982). Studies on the mechanism of chemosensitization by misonidazole *in vitro. Int. J. Radiat. Oncol. Biol. Phys.,* **8,** 705.

TANNOCK, I.F. (1980). The *in vivo* interaction of anti-cancer drugs with misonidazole or metronidazole: cyclophosphamide and BCNU. *Br. J. Cancer,* **42,** 871.

TWENTYMAN, P.R. (1981). Modification of tumour and host response to cyclophosphamide by misonidazole and WR 2721. *Br. J. Cancer,* **43,** 745.

TWENTYMAN, P.R. (1982). Growth delay in small EMT6 spheroids induced by cytotoxic drugs and its modification by misonidazole pretreatment under hypoxic conditions. *Br. J. Cancer,* **45,** 565.

TWENTYMAN, P.R., KALLMAN, R.F. & BROWN, J.M. (1979). The effect of time between X-irradiation and chemotherapy on the growth of three solid mouse tumours: I. Adriamycin. *Int. J. Radiat. Oncol. Biol. Phys.,* **5,** 1255.

TWENTYMAN, P.R., BROWN, J.M., GRAY, J.W., FRANKO, A.J., SCOLES, M.A. & KALLMAN, R.F. (1980). A new mouse tumour model system (RIF-1) for comparison of end-point studies. *J. Natl Cancer Inst.,* **64,** 595.

WORKMAN, P., LITTLE, C.J., MARTEN, T.R. & 4 others. (1978). Estimation of the hypoxic cell sensitizer misonidazole and its O-demethylated metabolite in biological materials by reversed-phase liquid chromatography. *J. Chromatogr.,* **145,** 507.

WORKMAN, P. & TWENTYMAN, P.R. (1982). Enhancement by electron-affinic agents of the therapeutic effects of cytotoxic agents against the KHT tumour. Structure-activity relationships. *Int. J. Radiat. Oncol. Biol. Phys.,* **8,** 623.

WORKMAN, P. (1979). Effects of pretreatment with phenobarbitone and phenytoin on the pharmacokinetics and toxicity of misonidazole in mice. *Br. J. Cancer,* **40,** 335.

WORKMAN, P. (1980). Pharmacokinetics of hypoxic cell radiosensitizers. A review. In *Radiation Sensitizers,* (Ed. Brady), New York: Masson, p. 192.


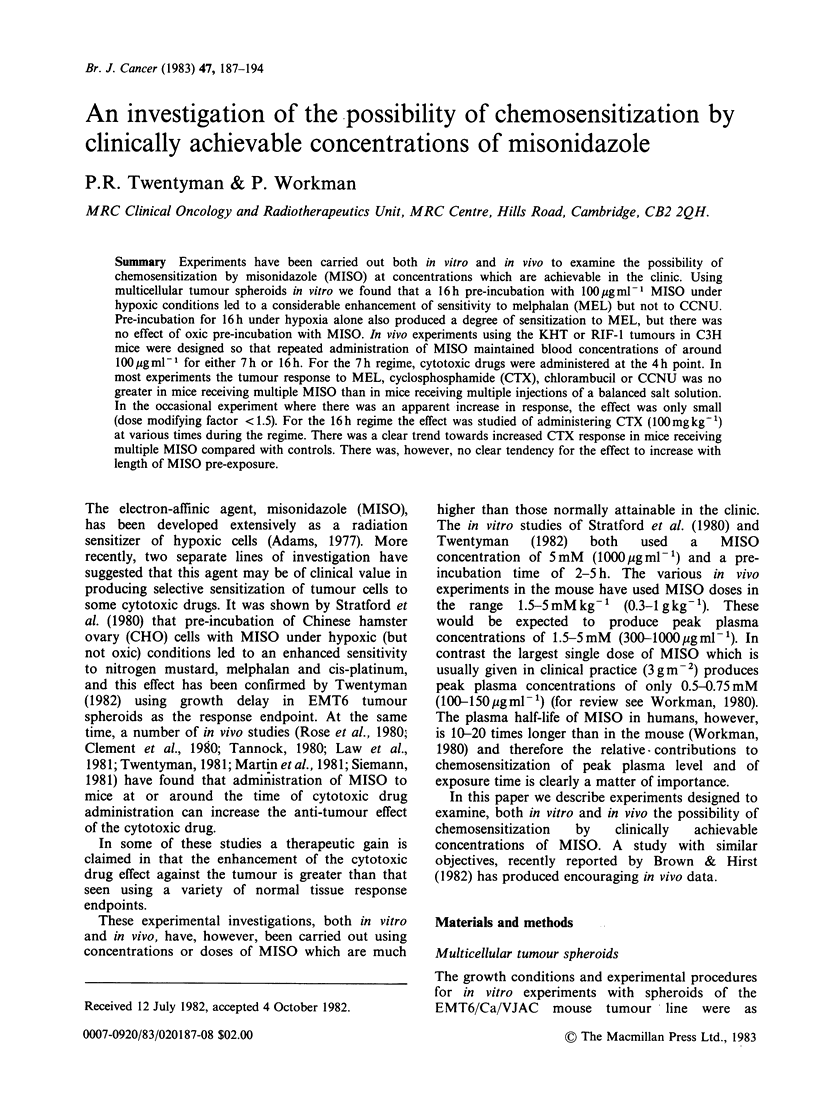

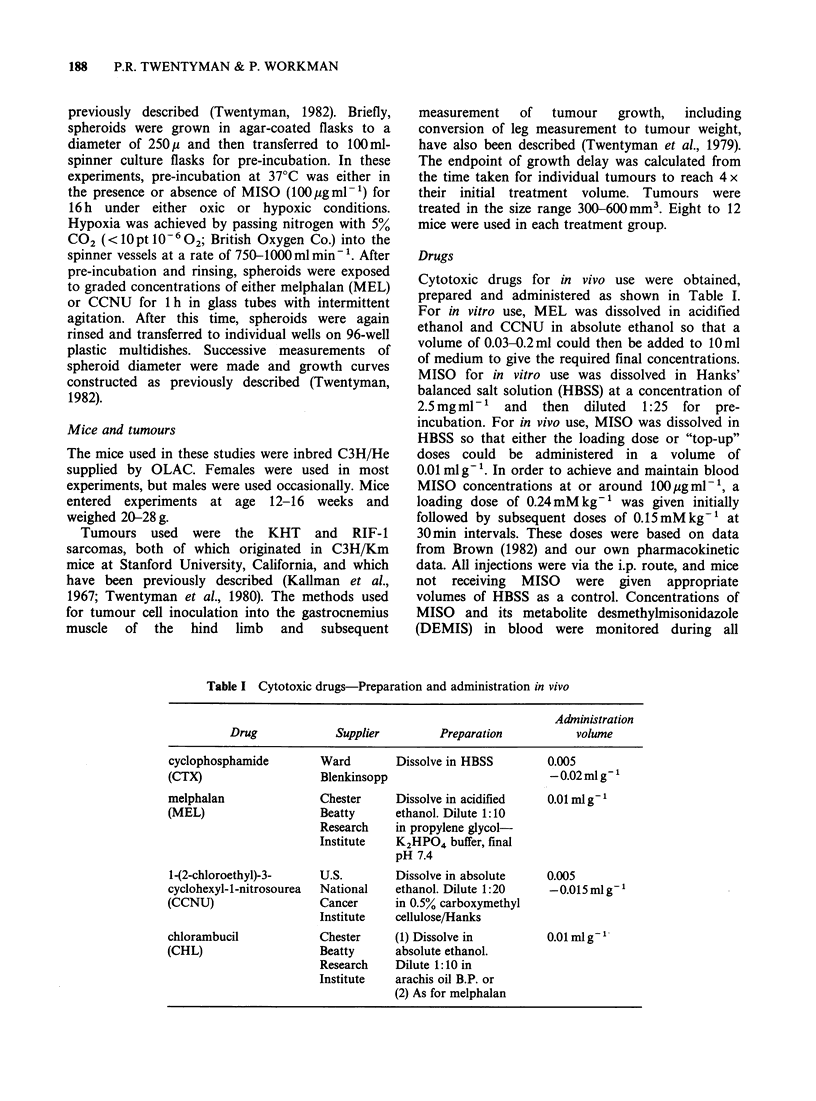

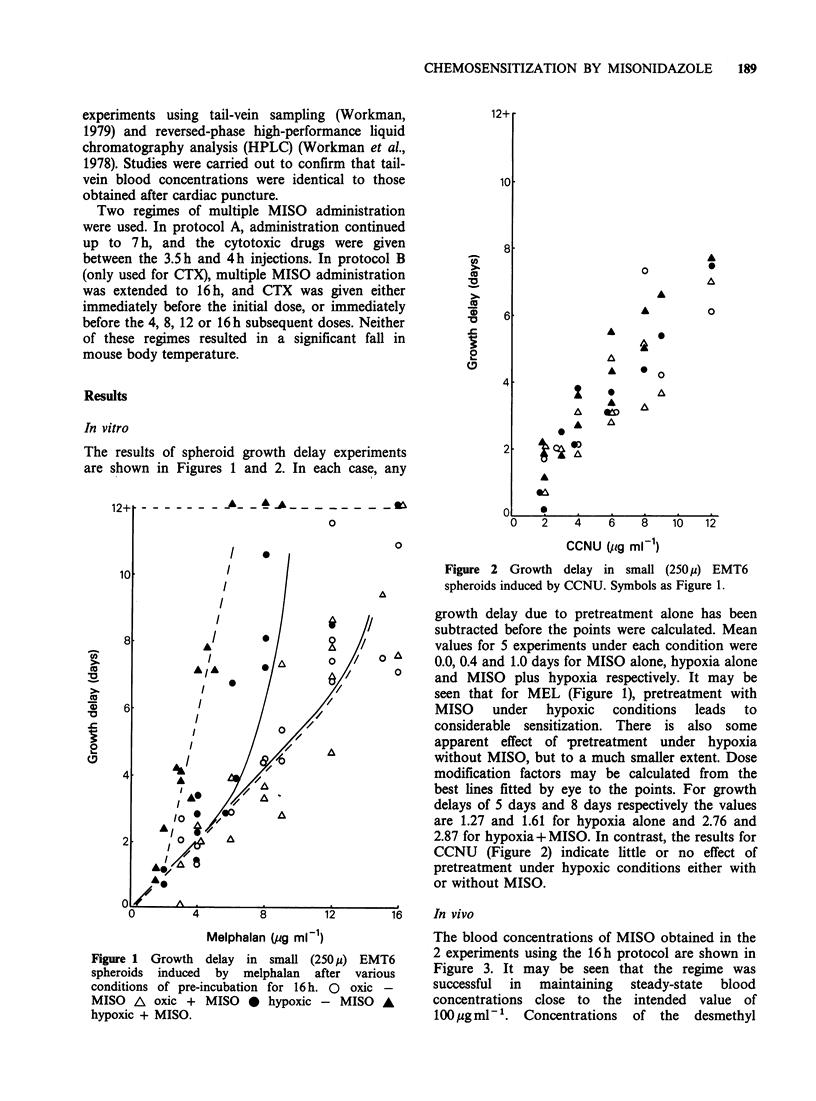

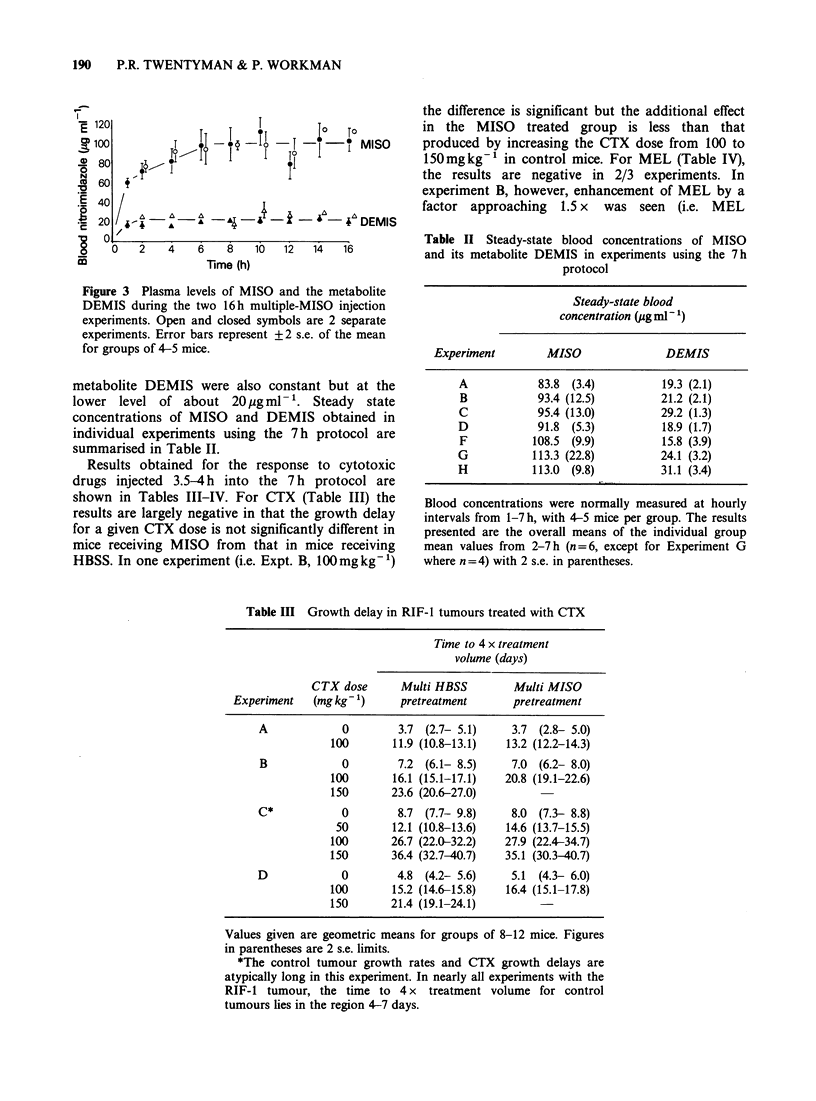

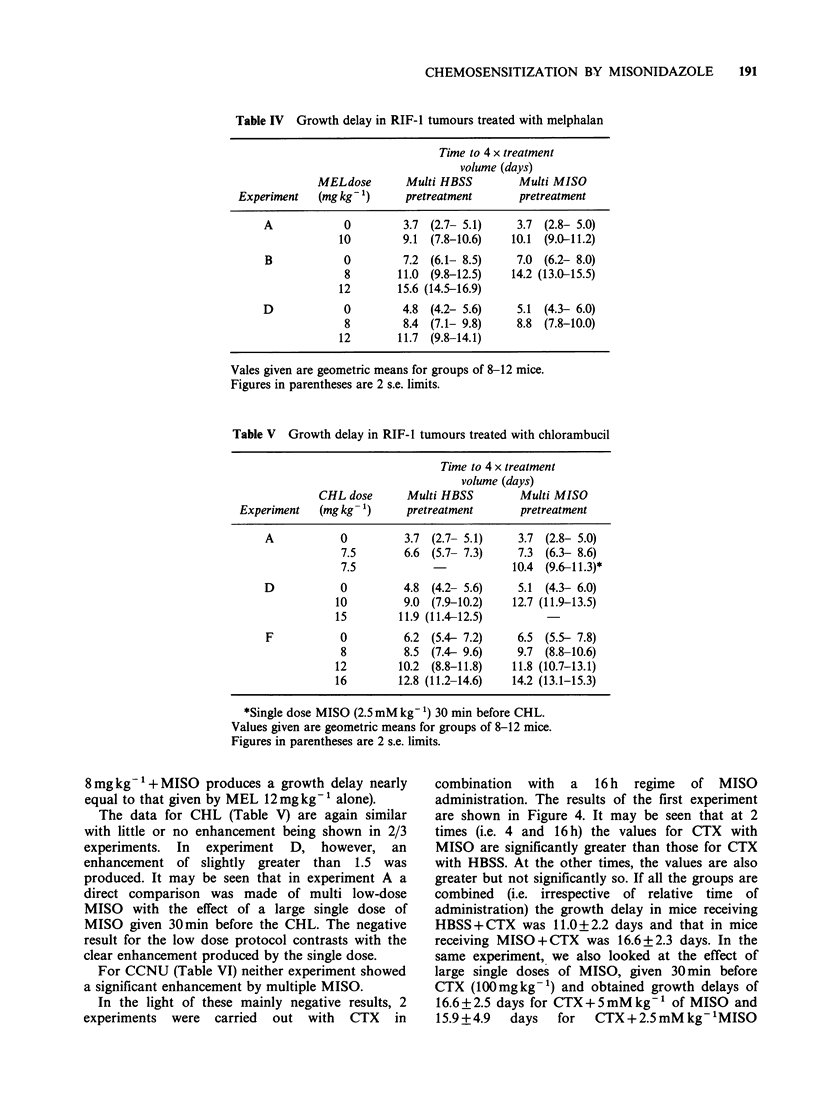

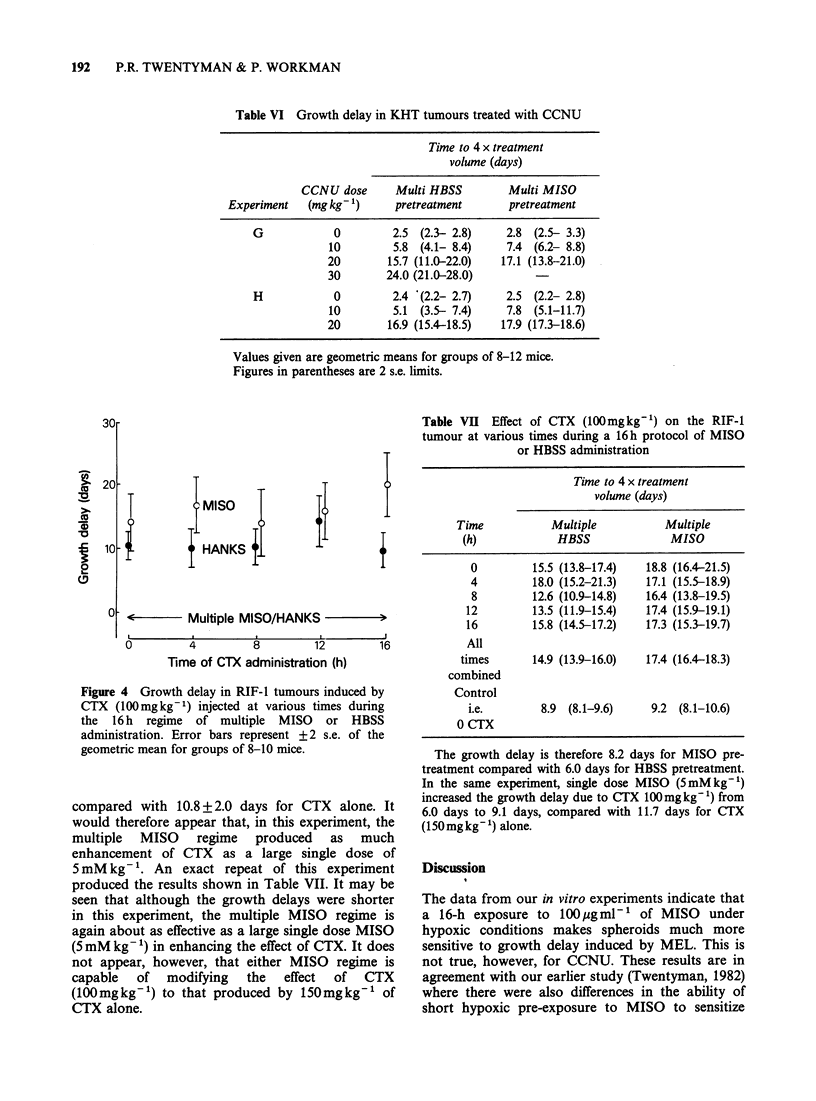

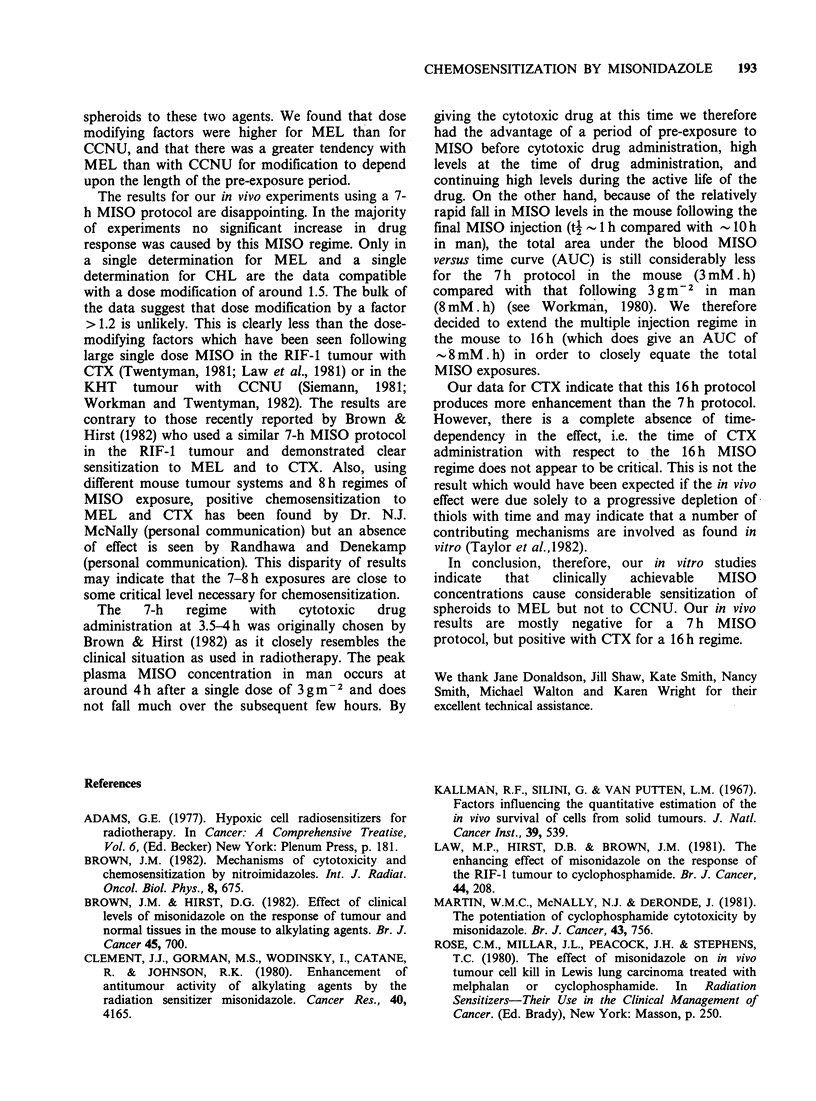

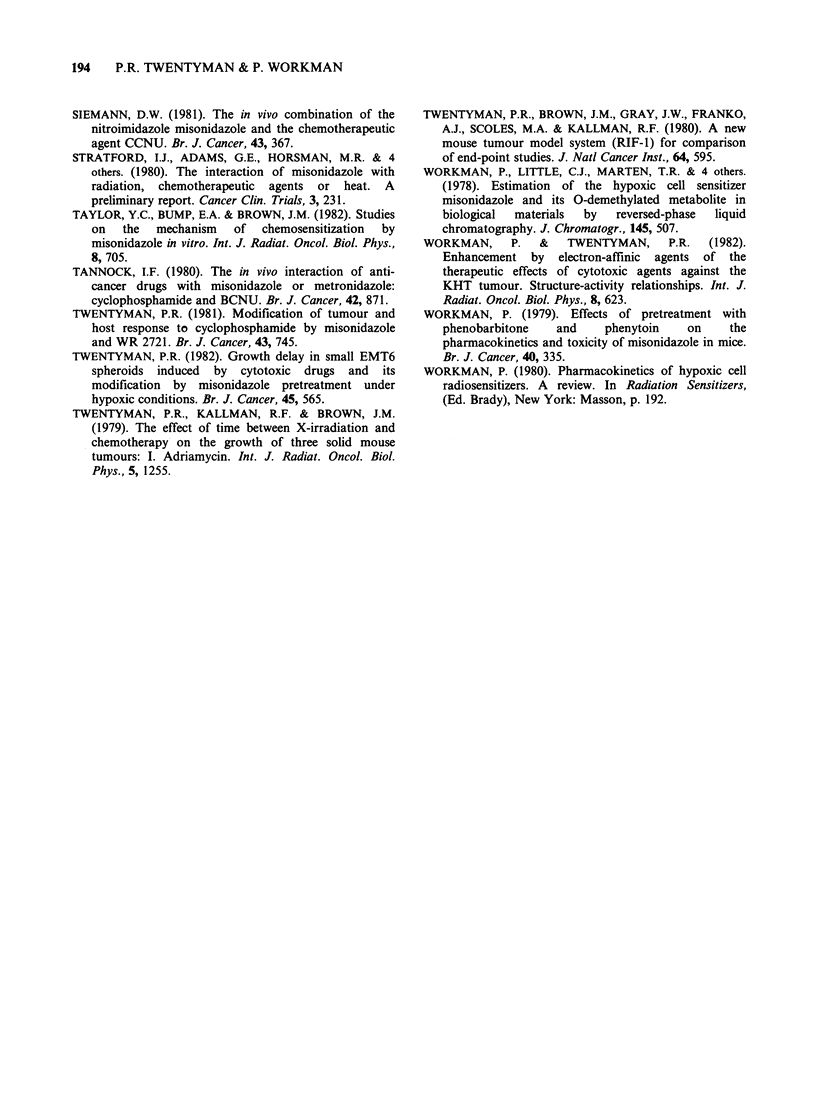


## References

[OCR_00713] Brown J. M., Hirst D. G. (1982). Effect of clinical levels of misonidazole on the response of tumour and normal tissues in the mouse to alkylating agents.. Br J Cancer.

[OCR_00708] Brown J. M. (1982). The mechanisms of cytotoxicity and chemosensitization by misonidazole and other nitroimidazoles.. Int J Radiat Oncol Biol Phys.

[OCR_00719] Clement J. J., Gorman M. S., Wodinsky I., Catane R., Johnson R. K. (1980). Enhancement of antitumor activity of alkylating agents by the radiation sensitizer misonidazole.. Cancer Res.

[OCR_00726] Kallman R. F., Silini G., Van Putten L. M. (1967). Factors influencing the quantitative estimation of the in vivo survival of cells from solid tumors.. J Natl Cancer Inst.

[OCR_00732] Law M. P., Hirst D. G., Brown J. M. (1981). Enhancing effect of misonidazole on the response of the RIF-1 tumour to cyclophosphamide.. Br J Cancer.

[OCR_00738] Martin W. M., McNally N. J., De Ronde J. (1981). Enhancement of the effect of cytotoxic drugs by radiosensitizers.. Br J Cancer.

[OCR_00753] Siemann D. W. (1981). In vivo combination of misonidazole and the chemotherapeutic agent CCNU.. Br J Cancer.

[OCR_00758] Stratford I. J., Adams G. E., Horsman M. R., Kandaiya S., Rajaratnam S., Smith E., Williamson C. (1980). The interaction of misonidazole with radiation, chemotherapeutic agents, or heat: a preliminary report.. Cancer Clin Trials.

[OCR_00770] Tannock I. F. (1980). In vivo interaction of anti-cancer drugs with misonidazole or metronidazole: cyclophosphamide and BCNU.. Br J Cancer.

[OCR_00764] Taylor Y. C., Bump E. A., Brown J. M. (1982). Studies on the mechanism of chemosensitization by misonidazole in vitro.. Int J Radiat Oncol Biol Phys.

[OCR_00793] Twentyman P. R., Brown J. M., Gray J. W., Franko A. J., Scoles M. A., Kallman R. F. (1980). A new mouse tumor model system (RIF-1) for comparison of end-point studies.. J Natl Cancer Inst.

[OCR_00780] Twentyman P. R. (1982). Growth delay in small EMT6 spheroids induced by cytotoxic drugs and its modification by misonidazole pretreatment under hypoxic conditions.. Br J Cancer.

[OCR_00786] Twentyman P. R., Kallman R. F., Brown J. M. (1979). The effect of time between X-irradiation and chemotherapy on the growth of three solid mouse tumors--I. Adriamycin.. Int J Radiat Oncol Biol Phys.

[OCR_00775] Twentyman P. R. (1981). Modification of tumour and host response to cyclophosphamide by misonidazole and by WR 2721.. Br J Cancer.

[OCR_00813] Workman P. (1979). Effects of pretreatment with phenobarbitone and phenytoin on the pharmacokinetics and toxicity of phenytoin on the pharmacokinetics and toxicity of misonidazole in mice.. Br J Cancer.

[OCR_00799] Workman P., Little C. J., Marten T. R., Dale A. D., Ruane R. J., Flockhart I. R., Bleehen N. M. (1978). Estimation of the hypoxic cell-sensitiser misonidazole and its O-demethylated metabolite in biological materials by reversed-phase high-performance liquid chromatography.. J Chromatogr.

[OCR_00806] Workman P., Twentyman P. R. (1982). Enhancement by electron-affinic agents of the therapeutic effects of cytotoxic agents against the KHT tumor: structure-activity relationships.. Int J Radiat Oncol Biol Phys.

